# Monte Carlo-based data generation for efficient deep learning reconstruction of macroscopic diffuse optical tomography and topography applications

**DOI:** 10.1117/1.JBO.27.8.083016

**Published:** 2022-04-28

**Authors:** Navid Ibtehaj Nizam, Marien Ochoa, Jason T. Smith, Shan Gao, Xavier Intes

**Affiliations:** Rensselaer Polytechnic Institute, Department of Biomedical Engineering, Troy, New York, United States

**Keywords:** diffuse optical tomography, diffuse optical topography, fluorescence molecular tomography, biophotonics, deep learning, Monte Carlo modeling

## Abstract

**Significance:**

Deep learning (DL) models are being increasingly developed to map sensor data to the image domain directly. However, DL methodologies are data-driven and require large and diverse data sets to provide robust and accurate image formation performances. For research modalities such as 2D/3D diffuse optical imaging, the lack of large publicly available data sets and the wide variety of instrumentation designs, data types, and applications leads to unique challenges in obtaining well-controlled data sets for training and validation. Meanwhile, great efforts over the last four decades have focused on developing accurate and computationally efficient light propagation models that are flexible enough to simulate a wide variety of experimental conditions.

**Aim:**

Recent developments in Monte Carlo (MC)-based modeling offer the unique advantage of simulating accurately light propagation spatially, temporally, and over an extensive range of optical parameters, including minimally to highly scattering tissue within a computationally efficient platform. Herein, we demonstrate how such MC platforms, namely “Monte Carlo eXtreme” and “Mesh-based Monte Carlo,” can be leveraged to generate large and representative data sets for training the DL model efficiently.

**Approach:**

We propose data generator pipeline strategies using these platforms and demonstrate their potential in fluorescence optical topography, fluorescence optical tomography, and single-pixel diffuse optical tomography. These applications represent a large variety in instrumentation design, sample properties, and contrast function.

**Results:**

DL models trained using the MC-based *in silico* datasets, validated further with experimental data not used during training, show accurate and promising results.

**Conclusion:**

Overall, these MC-based data generation pipelines are expected to support the development of DL models for rapid, robust, and user-friendly image formation in a wide variety of applications.

## 1 Introduction

Diffuse optical imaging (DOI) has found utility for many applications in biomedicine, including neuroscience, tissue physiologic monitoring, and diagnostics. DOI encompasses various modalities that leverage near-infrared light sensing or fluorescence-based contrast(s) to probe biotissue status. Of importance, biological samples imaged in DOI are intact, strongly diffusive tissues. Hence, DOI is typically characterized by the use of highly ill-posed light transport models. Two main modalities within DOI that often target macroscopic imaging of tissues are diffuse optical tomography (DOT)^1^ and topography (DOTP).^2^^,^^3^ While DOT aims to retrieve the full three-dimensional (3D) distribution of the target absorbers, DOTP only aims at retrieving their two-dimensional (2D) depth profiles. Hence, DOTP is better suited for applications where fast imaging over large fields of views is desired, while DOT can provide higher 3D spatial accuracy of the target. Despite their differences in output, DOT and DOTP recover the targets of interest within tissues through an inverse problem. This inverse problem is often linearized to reduce its ill-posedness.^4^ As for most DOI applications, the reconstruction accuracy is then dependent on realistically modeling photon transport within the sample and in the types of experimental or processing constraints enforced on the model. Thus, the image formation problem in DOI remains mostly an expert, lab-centric discipline.^4^^–^^6^ Though following ubiquitous trends in all the biomedical imaging fields, there has been a gradual interest in leveraging deep learning (DL) approaches to achieve the challenging DOT and DOPT computational task(s).^1^^,^^7^ DL’s use is appealing toward providing robust, user-friendly, ultrafast, and computationally efficient models that can replace the various inverse solving strategies currently used in DOI. Beyond ease of use, DL methods have been demonstrated to be orders of magnitude faster and correspondingly, better positioned for various biomedical applications that require “real-time” feedback.^7^

One main challenge in developing DL methodologies is the need for robust data generation workflows that can be applied to different types of DL architectures and imaging scenarios. Indeed, for DOT and DOTP, the lack of large publicly available data sets for network training is particularly striking. Most of the used training sets for proposed DOT and DOTP architectures rely on simplistic geometric structures such as spheres, which differs from reality as tumors and other tissue absorbers are not likely to be geometrically distributed. Such structures for training create the doubt whether networks will be able to generalize to more complex nongeometrical tumor distributions in experimental settings. Since enhanced EMNIST characters (introduced in Ref. 1) represent nongeometrical structures and can vary from number, symbols, etc., we estimate that these more complex shapes should result in more robust training. Also, the trained network would be able to generalize to reconstruct less complex shapes such as spheres and cylinders and yet be more prepared to tackle nongeometrical *in vitro* or *in vivo* 3D distributions.

In addition, given the wide variety of apparatus designs and configurations, obtaining well-controlled data sets for training and validation of DL workflows for these applications is a unique challenge. However, despite the differences, both DOT and DOTP traditionally rely on similar photon propagation modeling that can be leveraged to obtain a data generation workflow that works for both approaches. Herein, we propose a computationally efficient data generation workflow that relies on a single light propagation model of the homogeneous sample space, called $W_{\text{matrix}}$ (or Jacobian) across the paper, and the use of enhanced EMINST-based characters^1^ to generate nongeometric randomly distributed targets. This data-generation workflow is individually adapted for validation across three different macroscopic DOI applications and architectures: K-space reflectance DOT, hyperspectral structured light DOT, and lifetime-based DOTP. For simulation of the single homogeneous light propagation models, the open-sourced software “Monte Carlo eXtreme” (MCX) or “Mesh-based Monte Carlo” (MMC)^8^ is used.

Section 2 describes the general workflow of data generated using voxel (MCX) domain and mesh (MMC) domain-based approaches. The use of enhanced EMNIST characters is constant across approaches, but the simulation process of the $W_{\text{matrix}}$ is adjusted based on the illumination and detection. Furthermore, the simulation process adopts either a voxel or mesh-based approach. Section 3 details the simulation workflows along with their DL-based applications. The generated datasets are tested across three configurations: K-space illumination/widefield detection reflectance DOT, hyperspectral structured light transmission DOT, and widefield illumination/detection lifetime DOTP. DL workflows have been previously reported in Refs. 1 and 2 for K-space DOT and DOTP, respectively. However, we report the initial results herein for the mesh-based DOT approach. For K-space reflectance DOT and widefield DOTP, the application-specific DL workflows are validated *in silico* and experimentally in tissue-mimicking phantom samples. All resources used herein for data generation workflow (MATLAB), deep neural network (DNN) training (Tensorflow/Keras), and experimental validation are planned to be made available for the community. Altogether, our contributions aim to assist the community in adapting/incorporating open-sourced and experimentally representative voxel/mesh-based light propagation simulation routines for enhanced DL inverse solving potential.

## 2 Voxel and Mesh-Based Data Generation

### 2.1 MCX-Based Generation

MCX has rapidly emerged as an important MC-based framework for efficient generation of *in silico* DOT data.^8^ The voxel-based MCX workflow, although limited in resolution and accuracy compared to its mesh-based counterpart (MMC), is computationally less intensive, making it ideal for scenarios where speed is of the essence, such as in DL applications. MCX leverages the strong support provided by MATLAB and its numerous toolboxes for data visualization and processing. In addition, the built-in features of MCX, namely the ability to change the voxel size and the number of photons launched for MC, enable us to control the time needed to execute an MC-based workflow. This flexibility makes MCX suitable for different scenarios pertaining to DOT/mesoscopic FMT (MFMT) (depth range of a few millimeters). Traditional model-based DOT/MFMT reconstructions [for instance, least-squares (LSQ) and total variation minimization by augmented Lagrangian (TVAL)] are mainly achieved by accurately simulating the forward model on MCX to compute the Jacobian needed to solve the inverse problem.^4^ However, in the DL pipeline, the DOT/MFMT data in MCX can be generated either using the MCX-computed Jacobian or through direct MC simulation. The latter sacrifices speed in favor of a more accurate data-generation scheme. We have applied the first strategy to a K-space reflectance arrangement for fluorescence tomography (FT) and the second to a widefield transmission arrangement for DOT. In addition, the ability to incorporate biologically relevant fluorescence lifetime values into the MCX-based data generation scheme has been utilized for a unique widefield lifetime topography technique. These strategies are elaborated on in Sec. 3.

### 2.2 MMC-Based Generation

Despite the importance of a voxel-based data generation pipeline, a sample’s boundary conditions are better represented by mesh-based models as described in Ref. 8. The voxel (MCX)-based data generation pipeline is modified to generate simulated measurement vectors from mesh-based perturbed models. The generation of simulated datasets is made under the scheme of a single-pixel DOT setup reported in Ref. 9. It has been shown that single-pixel-based imaging can set experimental constraints like known wavelength channels and time-gates to further aid in the ill-posed DOT inverse solving process. Equation (1)^10^ explains a simplified mathematical model of the inverse problem to be solved, where single-pixel surface measurements b of the volume are acquired for wavelengths 1 to N:

$$[\begin{array}{c} b^{\lambda 1}\\ \vdots \\ b^{\lambda N} \end{array}]=[\begin{array}{ccc} W_{1}^{\lambda 1} & \cdots & W_{L}^{\lambda 1}\\ \vdots & \ddots & \vdots \\ W_{1}^{\lambda N} & \cdots & W_{L}^{\lambda N} \end{array}][\begin{array}{c} C_{1}\\ \vdots \\ C_{L} \end{array}].$$ (1)

To resolve the 3D spatial distribution of the absorbers, a complex Jacobian matrix is created. Of importance, due to the use of time-resolved data sets, structured light strategies, and potential application for preclinical applications, a flexible and accurate light propagation forward model is necessary. Hence, we employ MMC, a mesh-based Monte Carlo toolbox that enables the simulation of time-resolved data sets for samples with complex geometries that are illuminated by arbitrary wide-field light sources.^11^ The detailed MMC-based workflow and the potential for DL in mesh-based DOT reconstructions is discussed in Sec. 3.

## 3 Simulated Datasets for Deep Learning Applications

### 3.1 K-Space Reflectance Arrangement in MCX

The use of a reflectance geometry in MFMT is an attractive option since it allows exciting over a larger area while sustaining a relatively high illumination power.^12^ However, it has a low penetration depth and, in general, is not suitable for imaging deeply located fluorophores at conditions of high scattering. Such high-scattering conditions result in inaccurate reconstructions of 3D biodistribution by the traditional inverse solvers. However, with the advent of end-to-end DL-based approaches, some techniques have been developed, which have shown promise in reconstructing fluorescence distributions at relatively large depths with a high resolution.^7^^,^^13^

**Fig. 1:**
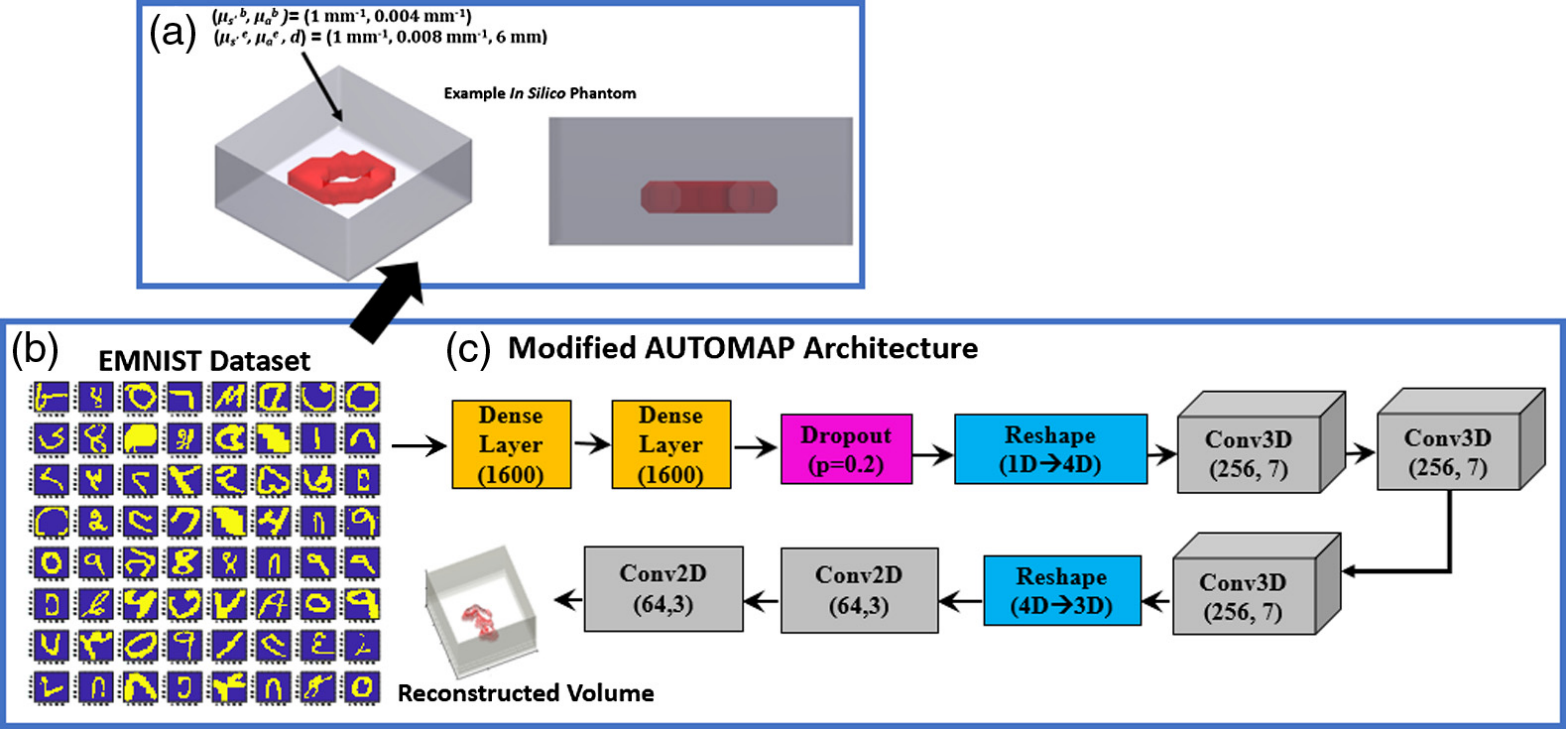
(a) An example *in silico* phantom (along with its side-on view) generated on MCX (with random OPs) from characters in the enhanced EMNIST dataset in (b). (c) The employed modified AUTOMAP (ModAM) network for carrying out reconstructions in k-space reflectance FT as well as widefield transmission diffuse optical tomography.

**Fig. 2:**
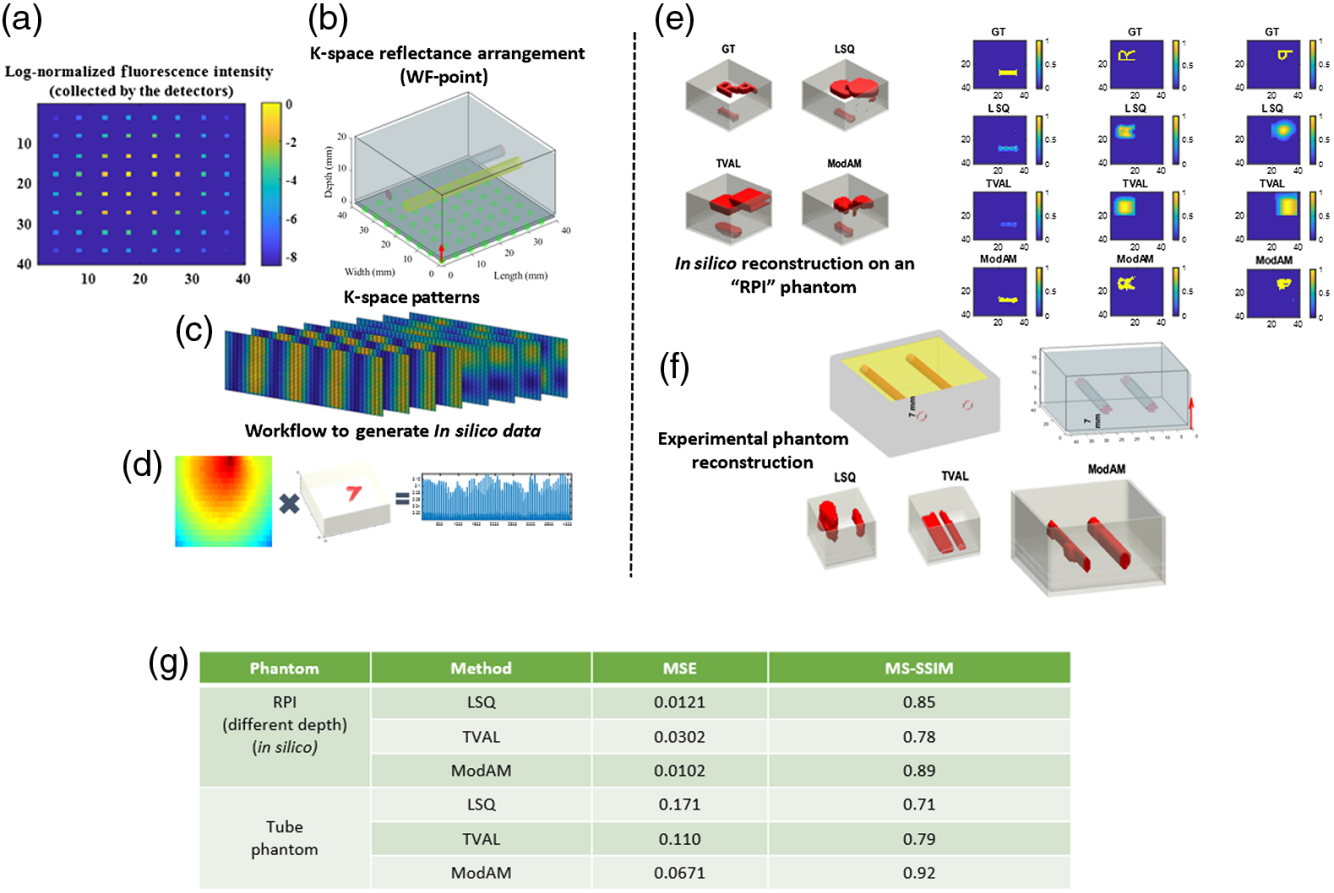
(a) The log-normalized fluorescence intensity collected by the 64 point detectors for the k-space (WF) illumination and point detection configuration (on MCX) as shown in (b). (c) Few of the 64 employed k-space patterns. (d) Rapid simulation workflow to generate the *in silico* data by multiplying a single Jacobian with different 3D volumes (containing fluorescent embeddings). (e) *In silico* results obtained from LSQ, TVAL, and ModAM along with the ground truth (GT) (in terms of the iso-volumes and the 2D cross-sections). (f) Experimental phantom reconstruction results using LSQ, TVAL, and ModAM. (g) Quantitative evaluation of the results (in terms of MSE and MS-SSIM) for the reconstructions shown in (e) and (f).

To this end, our group developed a CNN-based technique,^1^ utilizing a modified AUTOMAP (ModAM) architecture,^14^ as an end-to-end approach, to perform high-resolution 3D reconstructions for k-space reflectance fluorescence DOT. This work was the first known attempt at achieving 3D reconstructions in K-space reflectance FT. Herein, we detail how the MCX-based data generation scheme allows us to achieve high-quality reconstructions for a challenging optical tomography paradigm. As shown by the example in Fig. 1(a), MCX, along with its MATLAB packages, can be utilized to generate an *in silico* phantom. The optical properties (OPs) of the phantom can be varied over a wide range to adapt to the conditions of the imaging experiment. The *in silico* dataset is derived from an enhanced EMNIST database (composed of characters from different datasets such as EMNIST, Fashion MNIST, and Bengali.AI). Some of the characters from the enhanced EMNIST dataset are shown in Fig. 1(b), which has been previously demonstrated to be suitable for capturing the spatially complex biodistributions associated with different imaging scenarios, including *in vivo* imaging.^2^^,^^15^ The structure of the ModAM network is shown in Fig. 1(c). The choice of the AUTOMAP architecture stems from its initial development in Ref. 14, where the network was shown to be suitable for K-space sparse acquisition (as utilized in our work). In addition, the network was demonstrated to have a superior noise immunity, reducing reconstruction artifacts. The network was suitably modified for our tasks by adding additional convolutional and dropout layers. Following the MC simulation on MCX, the intensity obtained on the detector plane [as shown in Fig. 2(a) for 64 point detectors] can be translated to measurement vectors. Furthermore, as shown in Fig. 2(b), the optical geometry [in the source–detector (SD) configuration and hence, the plane of illumination/detection] can be considered for a reflection configuration, with the red arrow denoting the illumination and detection plane and the green dots representing the position of the point detectors.

Moreover, MCX can input different SD types (point, planar, and pattern, to name a few) to enable the modeling of different optical tomography paradigms. MCX is unique in its ability to simulate extended SD with any spatial distribution template. Hence, we can obtain the patterns generated experimentally using our gated-ICCD apparatus. The gated-ICCD system was designed for 2D and 3D wide-field imaging and leveraged structured light illumination. Digital micromirror devices (DMDs) are used to impart 64 illumination k-space patterns [some are shown in Fig. 2(c)]. Further details about the experimental setup can be found in Ref. 16. The experimental patterns are employed directly on MCX to compute the forward model (or the Jacobian) with a continuous wave formulation. In addition, we opt for an efficient data generation technique in which the Jacobian, calculated by MCX, is first produced and then multiplied with the embeddings (1 to 3) to generate the measurement vectors [shown in Fig. 2(d)]. This large *in silico* dataset is used to train the ModAM network. The details of the dataset and the network training/validation parameters are presented in Table 1. In the table, $\delta \mu_{a}$, $\mu_{s}$, and g stand for the difference between the absorption coefficient of the embedding(s) and the background, the scattering coefficient, and the anisotropy factor, respectively.

**Table 1:** Details of the training dataset and the modified AUTOMAP network for k-space reflectance FT (GPU: NVIDIA GeForce RTX 2080Ti).

Size of dataset	250,000 (1, 2, or 3 embeddings)
OPs	$\delta \mu_{a}=0.008$ to $0.2\;{\mathrm{mm}}^{-1}$
	Depth = 2 to 16 mm
	$\mu_{s}=10\;{\mathrm{mm}}^{-1}$
	g=0.90
	No. of photons (in simulation) = $10^{9}$
Time to generate dataset	375 min
Training/validation split	80/20
Batch size	32
No. of epochs	740
Loss function	MSE
Optimizer	Adam
Learning rate	$10^{-5}$
Training time	∼15 h

In Fig. 2(e), we display the reconstruction performance on an *in silico* phantom having three inclusions, “R,” “P,” and “I” at depths of 2, 8, and 14 mm, respectively. None of these embeddings are part of the original dataset. Compared with related works in the literature, this is the most spatially complex phantom ever reconstructed. The reconstructions obtained from ModAM (both in terms of the iso-volume and the 2D cross-sections) outperform the results obtained from the traditional LSQ and TVAL-based techniques visually. Quantitatively, the reconstruction results evaluated in terms of the mean-squared error (MSE) and the multiscale structural similarity index (MS-SSIM) are shown to outperform the traditional techniques as well [as seen from the table in Fig. 2(g)] even at higher depths (where the photons have been highly scattered).

Furthermore, the flexibility of MCX in replicating the experimental phantom conditions while computing the Jacobian meant that the network, trained entirely on *in silico* data, could be utilized effectively for an experimental phantom as well. A homogeneous phantom is created for the experiment with two NIR fluorescent capillaries (each of diameter 3 mm) embedded at a depth of 7 mm from the illumination and detection plane. The $\mu_{a}$, and reduced scattering coefficient, $\mu_{s}^{\prime }$, values of the homogeneous background (0.004 and $1\;{\mathrm{mm}}^{-1}$, respectively) are generated using a suitable mixture of agar, ink, and intralipid. The reconstruction results are shown in Fig. 2(f). It is clear, visually, that both LSQ and TVAL fail to reconstruct the shape and position of the extended tubes accurately. However, owing to the spatial heterogeneity present in our training dataset, the proposed ModAM network can reconstruct the tubes with higher accuracy than the traditional techniques. Also, the ability to reconstruct extended objects, such as tubes, is a significant advantage offered by our enhanced EMNIST-based data-generation scheme compared with similar works in the literature employing DL.^13^^,^^17^ The other DL-based reconstruction techniques mainly employ simple geometric structures (such as spheres) for training and testing. These observations are backed up by the quantitative results shown in Fig. 2(g). Moreover, there is a huge gain in computational efficiency (the network takes only 1 s for ∼200 reconstructions while LSQ and TVAL take ∼25 and 40 min for each reconstruction, respectively). Therefore, the proposed MCX-based DL workflow promises high-resolution 3D reconstructions in preclinical scenarios utilizing MFMT.

### 3.2 Widefield Transmission Geometry in MCX

Transmission configurations in tomography allow for higher penetration and hence, enable better reconstruction of contrasts embedded at higher depths.^18^ The use of widefield illumination and detection in conjunction has been shown to provide a wider field of view (FOV) for such reconstructions.^19^ As eluded to previously, MCX may be deployed to generate perturbed (ϕ) and unperturbed measurements($\phi_{0}$) directly from the MC simulation without explicitly computing the Jacobian. Generating the measurement vectors from the MC simulations [and not by multiplying a single Jacobian with the *in silico* embedding(s)] is a slow but more accurate process since it takes into account the variation of the OPs of the embeddings (in the form of the perturbed measurement vector). This approach is adapted to carry out initial investigations for a DL-based $\delta \mu_{a}$ reconstruction workflow in transmission DOT, which employs a widefield illumination-widefield detection configuration.

**Fig. 3:**
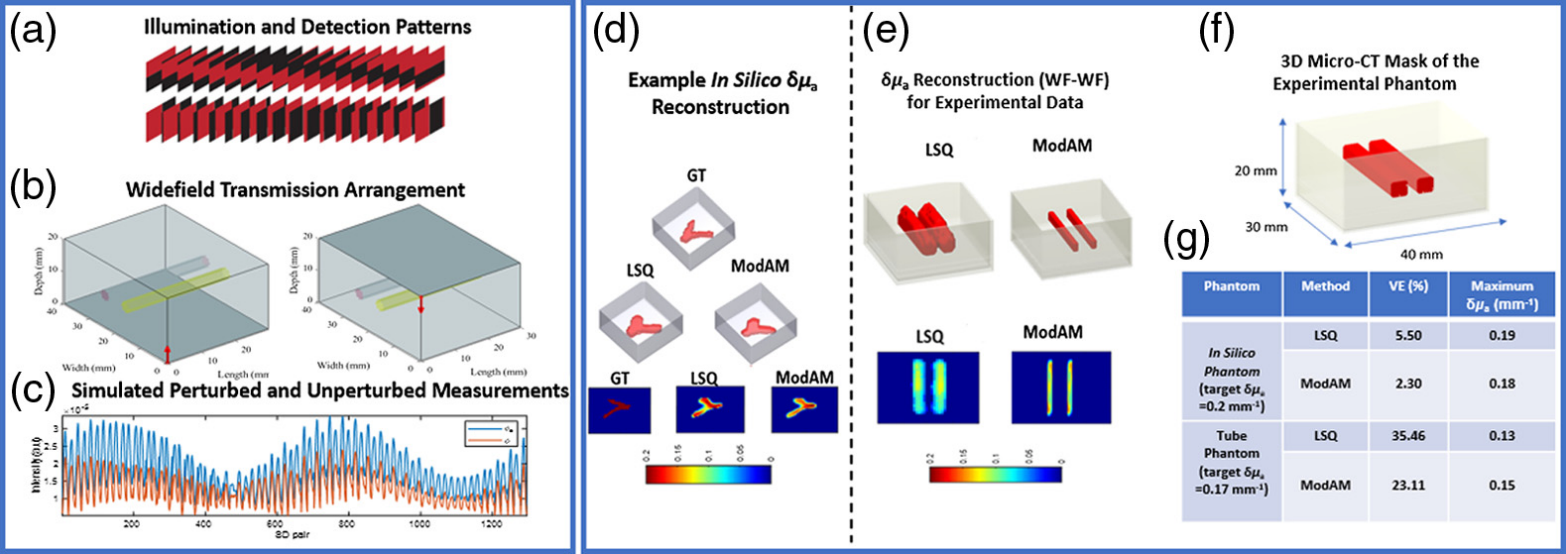
(a) 36 illumination and detection bar patterns used for the widefield illumination and detection configuration in transmission, as shown in (b) for MCX. (c) A pair of representative simulated unperturbed ($\phi_{o}$, in blue) and perturbed measurement vectors (ϕ, in red). (d) An example *in silico* reconstruction using LSQ and ModAM (in terms of the iso-volumes and 2D cross-sections). (e) Experimental phantom reconstruction using LSQ and ModAM. In both (d) and (e), the colorbar represents the reconstructed $\delta \mu_{a}$ values. (f) Micro-CT mask used as the GT for evaluating the VE for the experimental phantom. (g) Qunatitative results for the reconstructions shown in (d) and (e) in terms of the VE and the maximum reconstructed $\delta \mu_{a}$ value.

A similar DL architecture approach to the k-space reflectance tomography work in Sec. 3.1 is utilized in this work. The same EMNIST dataset and the ModAM network [Figs. 1(b) and 1(c)] are used for generating the *in silico* phantom and 3D $\delta \mu_{a}$ reconstructions, respectively. However, in this work, simple bar patterns [as shown in Fig. 3(a)] are used (experimentally acquired from our hyperspectral system) for illumination and detection. The hyperspectral system is equipped with DMDs for projecting both illumination and detection patterns. In addition, in this system, the measurement vector is obtained directly from the corresponding wavelength channel from a 16-channel photomultiplier tube detector. For more details regarding the apparatus and imaging protocol, the interested reader is referred to Ref. 19. The corresponding widefield–widefield transmission setup on MCX is shown in Fig. 3(b). Next, we carry out two separate MC simulations on MCX. An MC simulation with no embedding(s) generates $\phi_{0}$ while the one with the embedding produces ϕ. A sample pair of unperturbed and perturbed measurement vectors are displayed in Fig. 3(c) (in blue and red, respectively). A single Rytov-normalized measurement vector is produced from these measurements ($\log\;\frac{\phi_{0}}{\phi }$). In this way, a large dataset of measurement vectors is again generated for training and validation of the modified AUTOMAP network. The details of the network are displayed in Table 2.

**Table 2:** Details of the training dataset and the modified AUTOMAP network for widefield DOT (GPU: NVIDIA GeForce RTX 2080Ti).

Size of dataset	250,000 (1, 2, or 3 embeddings)
OPs	$\delta \mu_{a}=0.008$ to $0.2\;{\mathrm{mm}}^{-1}$
	Depth = 2 to 16 mm
	$\mu_{s}=10\;{\mathrm{mm}}^{-1}$
	g=0.90
	No of photons (in simulation) = $10^{7}$
Time to generate dataset	∼30 h
Training/validation split	80/20
Batch size	32
No. of epochs	795
Loss function	MSE
Optimizer	Adam
Learning rate	$10^{-5}$
Training time	∼16 h

It can be observed from the table that the parameters are identical to the ones shown in Table 1 except for the time required to generate the dataset (∼30  h instead of 375 min in Table 1). The significant increase in dataset-generation time highlights the trade-off between accuracy and speed. Of course, there is also a change in the number of epochs and training time because of a change in the nature of the measurement vectors. In addition, to decrease the time required to generate the dataset to something more manageable, the number of photons for the forward simulation is reduced to $10^{7}$ from $10^{9}$ (as seen in Table 1). We demonstrate in Figs. 3(d) and 3(e), the reconstruction performances of the ModAM network on a representative *in silico* phantom and an experimental phantom, respectively, compared with a traditional regularized LSQ-based technique. In the *in silico* case, the target for the reconstructed $\delta \mu_{a}$ value in the embedding is $0.2\;{\mathrm{mm}}^{-1}$ while that in the experimental phantom is $0.17\;{\mathrm{mm}}^{-1}$. The preparation of the experimental phantom follows the same protocol as discussed in the previous section, except that the tubes are now filled with an absorption contrast (instead of fluorophores) having a higher $\mu_{a}$ value compared with the homogeneous background (to create a $\delta \mu_{a}$ of $0.17\;{\mathrm{mm}}^{-1}$). Again, the network, trained entirely on *in silico* data, is used for both *in silico* and experimental phantom reconstructions. It is clear from the figures that the reconstruction performance of the ModAM network is superior compared with LSQ. In addition, we quantitatively evaluate the reconstruction performance in terms of volume error (VE) and the maximum reconstructed $\delta \mu_{a}$ value. For computing the VE, we use the GT as the reference volume for the *in silico* case, and the 3D micro-CT mask [obtained from an MARS photon-counting micro-CT scanner, shown in Fig. 3(f)] for the experimental phantoms. From the results tabulated in Fig. 3(g), ModAM outperforms LSQ for both *in silico* and experimental phantom reconstructions in terms of the VE and the maximum $\delta \mu_{a}$ reconstructed (closer to the target for ModAM compared with LSQ). Therefore, this initial study demonstrates great potential in correctly reconstructing the absorption contrast, an important biomarker in clinical scenarios, such as breast cancer imaging.

### 3.3 MCX-Based Widefield Lifetime Topography

In applications where simplicity in the imaging protocol is critical, such as in fluorescence-guided surgery, reflectance geometry configurations are desirable. However, to date, most fluorescence imaging systems are limited to 2D subsurface sensing of intensity information. Given that intensity readouts are dependent on many factors (fluorophore concentration, depth of inclusion, tissue OP, etc.), they are currently intrinsically limited in their utility. In contrast, topographic imaging (i.e., 2D depth resolved imaging) of fluorescence can be performed in reflectance geometry over large FOVs using widefield time-resolved cameras.^2^^,^^3^ In addition, microenvironmental information that can be gleaned from fluorescence lifetime (e.g., pH,^20^^,^^21^ temperature,^22^ protein–protein interactions,^23^ etc.) and can be utilized for enhanced insight. For this, the diffuse transport response [Eq. (2)] must be modeled. Using MCX, along with an experimentally representative instrument response function (IRF), one can simulate these data:^24^

$$\phi_{fl}(r_{s},r_{d},t)=[\int \eta (r)\int_{0}^{t}e^{-(t-t^{\prime })/\tau (r)}dt^{\prime })\int_{0}^{t^{\prime }}G_{r_{d}-r}(t^{\prime }-t^{\prime \prime })*G_{r-r_{s}}(t^{\prime \prime })dt^{\prime \prime })d^{3}r]*\mathrm{IRF}(t),$$ (2)

where $r_{s}$ and $r_{d}$ correspond to the SD positions, τ to the fluorescence lifetime (assumed monoexponential herein), η(r) to the fluorophore’s effective quantum yield distribution, and $G_{\left|r\right|}(t)$ to the MCX-computed propagators (semi-infinite boundary conditions and homogenous background OP). This inverse problem is normally constrained with priors, such as knowledge of the fluorescence lifetime^3^ and/or separate quantification of OP information.^2^^,^^3^ However, the fluorescence lifetime of many fluorophores depends on many factors, especially in biological systems, which cannot always be assumed constant throughout the sample of interest. Further, the effective η(r) is dependent on fluorescence concentration, which is difficult to ascertain *in vivo*.

For this, MCX can generate experimentally representative data over a large range of conditions, such as biologically relevant OP ranges, fluorescence lifetimes, and fluorophore concentrations. Importantly, using these data to train a DNN, one can map time-resolved fluorescence information coupled with OP information (e.g., retrieved through spatial frequency-domain imaging) directly to lifetime and depth information.^2^

**Fig. 4:**
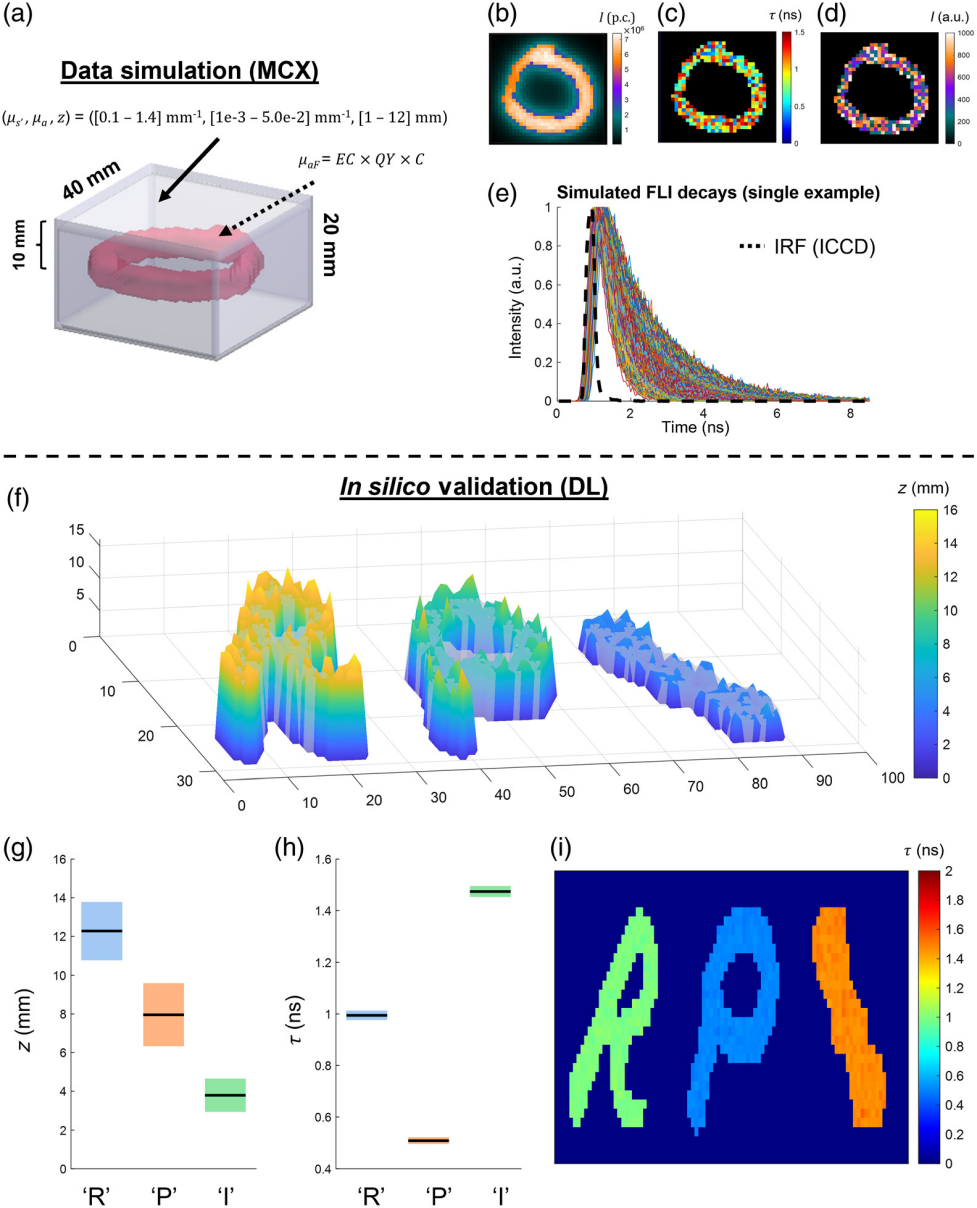
MCX simulation workflow for macroscopic FLI topography and in silico validation. (a) Example MNIST letter embedding in homogeneous phantom. Ranges of parameters used for training are given (further detailed in Table 1). (b) Example CW image obtained through MCX generation of embedding → detector light propagation. (c) Spatially independent lifetime and (d) scalar multiple for Poisson noise-based SNR variation. (e) Example TPSFs generated for a single data voxel. The camera (gated-ICCD) IRF is overlaid. (f) Retrieved depth profile from letter embeddings, (g) boxplot for retrieved depths at each letter, (h) boxplot for retrieved lifetimes at each letter, and (i) the 2D lifetime reconstruction.

The simulation workflow [shown in Figs. 4(a)–4(e)] will be summarized in brief below. In a similar fashion to Fig. 1, a binary figure is chosen at random as a fluorescence embedding for each simulated data iteration. A set of parameters are then chosen at random to set the conditions of the propagation model (provided in Table 3).

**Table 3:** Details of the training dataset and the Siamese DNN used for widefield fluorescence lifetime topography (GPU: NVIDIA GTX 3090).

Size of dataset	5000 (1 embedding)
OPs	$\mu_{a}=0.001$ to $0.05\;{\mathrm{mm}}^{-1}$
	Depth = 1 to 12 mm
	$\mu_{s^{\prime }}=0.1$ to $1.4\;{\mathrm{mm}}^{-1}$
	No. of photons (in simulation) = $10^{6}$
Time to generate dataset	∼1 h
Lifetime	τ=0.3 to 1.5 ns
Fluo. concentration	C=1 to 100 μM
Training/validation split	75/25
Batch size	32
No. of epochs	75
Loss function	MSE
Optimizer	Adam
Learning rate	$10^{-5}$
Training time	∼15 min

For the homogeneous background OPs, a single value of $\mu_{a}$ and $\mu_{s^{\prime }}$ is set herein. Though depth ranging from shallow (1 mm) to exceedingly deep (12 mm) are used at random throughout data generation, the depth of embedding (enhanced EMNIST, explained previously) is set as a constant for each data instance for simplicity. Widefield reflectance imaging geometry is set by independently simulating the source → embedding and embedding → detector Jacobians/Green’s functions for accuracy. Given that fluorescence lifetime can often vary from pixel to pixel, spatially independent values for lifetime were set [e.g., Fig. 4(c)] to ensure robustness in the trained model’s lifetime inference. Additional model robustness was ensured by pixelwise Poisson noise variation, set by multiplication with a scalar intensity value before max normalization [Fig. 4(d)]. An example set of temporal point spread functions (TPSFs) simulated through one iteration of this process, as well as the system IRF, is shown in Fig. 4(e).

Validation *in silico* was undertaken by simulation of a rectangular prism block phantom including three embeddings: letters “R-, “P,- and “I-. Each embedding was assigned a different depth and lifetime value (“R” = [12 mm, 1.0 ns], “P” = [8 mm, 0.3 ns], and “I” = [4 mm, 1.5 ns]). The phantom OPs were set to $\mu_{s^{\prime }}=1\;{\mathrm{mm}}^{-1}$ and $\mu_{a}=0.01\;{\mathrm{mm}}^{-1}$. Figure 4(f) shows the depth profiles obtained across the entire volume as well as the GT (gray transparency plane). Figures 4(g) and 4(h) show the lifetime and depth values retrieved for each letter embedding. Both lifetime and depth maps obtained via DL are in high concordance with the GT (${\mathrm{MSE}}_{\tau }<0.0001$ for lifetime and ${\mathrm{MSE}}_{z}<0.50$ for depth).

**Fig. 5:**
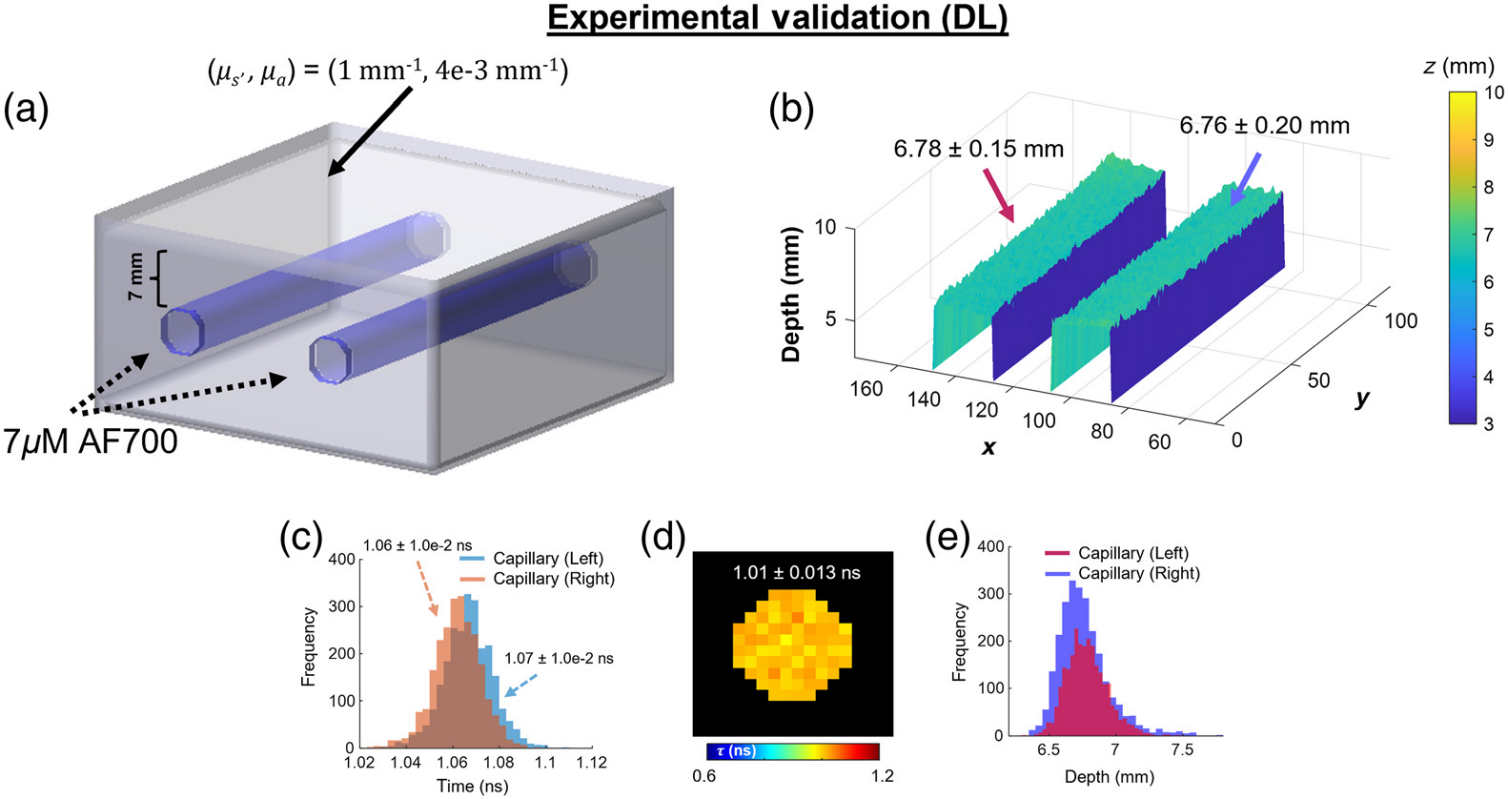
Macroscopic FLI topography via DNNs trained with MCX-simulated data: experimental validation. (a) Experimental homogeneous phantom containing two fluorescence embeddings (AF700). (b) 2D depth profiles and average ± standard deviation retrieved through the DNN for both capillaries. (c) Histogram of lifetime values obtained through the trained DNN. (d) Lifetime results of pure dye imaged with a separate, time-resolved SPAD camera. (e) Depth results from (b) displayed in histogram form.

Our gated-ICCD apparatus performed experimental validation by time-resolved imaging of a homogeneous phantom containing two fluorescent capillaries. The agar phantom [Fig. 5(a)] was prepared in similar fashion as previously described ($\mu_{s^{\prime }}=1\;{\mathrm{mm}}^{-1}$ and $\mu_{a}=0.004\;{\mathrm{mm}}^{-1}$, respectively). Two glass capillaries, located at 7 mm depth below the phantom surface, were filled with 7  μM solution of AlexaFluor 700. The excitation wavelength was set to 700 nm with 1.5 mW measured at the imaging plane. Imaging over 226 time-gates was performed using 40 ps gate-step, 300 ps gate-width, 353 ms exposure time, hardware binning of 2×2, and 450 mV MCP gain voltage. An emission filter (Semrock, FF01-715LP) was used for fluorescence imaging. Herein, micro-CT data were registered to the ICCD imaging plane and used to mask the capillaries at their correct x and y coordinates. A single AF700 well (300 nM concentration) was also imaged using a separate mesoscopic time-resolved SPAD imager, for validation Fig. 5(d).

A dual-input DNN architecture based on FLI-NET (explained elsewhere^2^) was trained on 5000 data instances for 75 epochs (≈15  min using NVIDIA GTX 3090) using *Tensorflow* with *Keras* backend. Afterward, the model’s input dimensionality was changed to accept the spatial dimension of the experimental data, and weights obtained through training were reloaded—a step only made possible due to the fully convolutional nature of the DNN used herein. OP were set to that used when building the phantom, and the MFLI data were max-normalized equivalently to that used in the data simulation workflow [see Fig. 4(e)].

Though the model was trained using a wide range of parameters, the experimental results show high agreement with ground-truth for both lifetime of AF700 [pure dye ≅1.01  ns, Fig. 5(c)] and depth [Figs. 5(b) and 5(e)]. Indeed, by training the model using only MCX-simulated data, the DNN-retrieved depth within 0.5 mm (absolute percentage error of 3.19±2.9%) and lifetime within 0.05 ns (absolute percentage error of 5.74±1.0%) of the known/independently validated results. In addition, as expected, the values (lifetime and depth) obtained across both capillaries are in high concordance.

### 3.4 MMC-Based Widefield DOT

Under the MMC environment, one Jacobian matrix is computed using the adjoint method and simulated at each wavelength 1 to N for absorber numbers 1 to L. Together with the modeling of these Jacobians and time gates, the spatial distribution of the absorption contrast C can be retrieved for absorbers 1 to L.

**Fig. 6:**
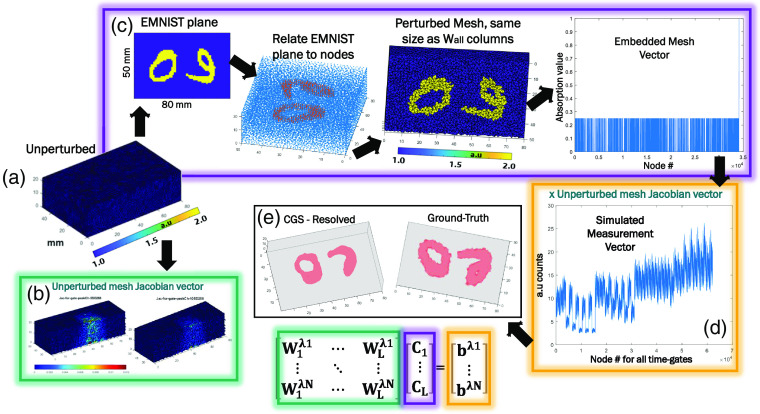
(a) Unperturbed node volume used in MMC. (b) Illumination and detection pair 1 as calibrated with the hyperspectral single-pixel DOT platform and an external CCD. (c) Example of TPSF for TD Jacobian for node 1. (d) Examples of light propagation through Jacobian for SD pair at three time gates. (e) Summary of size of $W_{\text{matrix}}$ containing multiwavelength and multitime-gate information.

The generated unperturbed mesh shown in Fig. 6(a) is used for the MMC photon propagation modeling with $10^{9}$ photons, which includes 36 sources and 36 detectors simulated in a transmission geometry following the bar patterns reported by Refs. 9 and 10 and already shown in Fig. 3(f). Rytov normalizations are accounted in the time domain (TD) Jacobian matrix formation with the Green’s functions outputted by MMC.^8^ Jacobians were retrieved for fixed selected time-gates estimated from the convolution of the SD pair configurations with IRFs. Fixed time-gates were selected at 50% rise of the TPSFs, at the max (Peak) value, 80% and 60% of the decay curve. Examples of the Jacobian matrices for two different spectral channels are displayed in Fig. 6(b) for the 288th SD pair. These Jacobians are then concatenated to form the matrix $W_{1}^{\lambda 1}$ to $W_{L}^{\lambda 1}$ as displayed in Eq. (1) for 12 λN spectral channels.

Once the concatenated Jacobian matrix is ready, a mesh with the same nodes and elements is perturbed with values representing enhanced EMNIST characters and a random absorption contrast. To perturb the node centroids, an enhanced EMNIST-based figure is resized and centered on matching the x and y dimensions of the unperturbed mesh. Later the coordinates of the non-zero elements in the 2D image are defined and repeated along the depth dimension resulting in (x,y,z) coordinates for the embedded element. Subsequently, a K-nearest neighbor algorithm with Minkowski distance metric with an exponent of 5 and finding five nearest neighbors were used to correlate the 2D enhanced EMNIST plane exemplified in Fig. 6(c) to the unperturbed mesh of size 34,202×1, resulting in a perturbed mesh with defined depth and enhanced EMNIST figure-based shape. An example of the correlated node mesh is shown in Fig. 6(c), where, by retrieving the position of the embedding with absorption value ($\mu_{a}$), the embedded mesh vector is obtained as shown in Fig. 6(c). The display of this vector in isovolume form is shown in the GT of Fig. 6(e).

Finally, the simulated single-pixel-based hyperspectral time-gated vector is generated by combining the embedded mesh vector and concatenated matrix as shown in Figs. 6(b) and 6(c) for 1296 SD pairs, 4 time gates, and 12 wavelength channels. An example of this simulated measurement vector is shown in Fig. 6(d). To validate the correct simulation of the measurement vector, this one was used with the concatenated Jacobian matrix to inverse solve the 3D distribution of the embeddings. The inverse solving was done using conjugate gradient square inverse solver with a tolerance of $5\times 10^{-10}$ and 500 maximum iterations. Results for this reconstruction are shown in Fig. 6(e). This routine has been extended to simulate 20,000 enhanced EMNIST samples of which the measurement vectors provide similar quality reconstructions to the example herein provided.

Following the mesh-based data generation pipeline, the simulated measurement vector and GT embedded mesh vector were then input to an MLP-based architecture. Similar MLP architectures have been employed for DOT tasks in Refs. 25 and 26. Similar to the architectures presented throughout this work, the network aims to directly retrieve the absorption values and spatial distributions of the targets within the scattering media from the raw measurements. In this regard, the main differences rely on using a mesh-based approach and measurement vectors containing both time and spectral dimensions. As mentioned in Refs. 7, 9, 10, 27 to 29 and as summarized in Eq. (1) the incorporation of spectral and time dimensions into the ill-posed inverse problem adds more known variables to the ill-posed inverse problem, allowing for more accurate reconstruction of the optical parameters and chromophore concentrations. The measurement vectors were generated accounting following the arrangement of the same instrument used in Fig. 3.

**Fig. 7:**
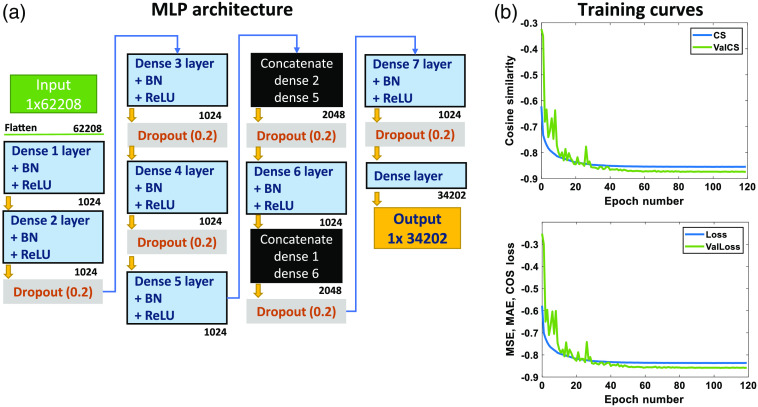
(a) MLP trained with MMC-simulated data. (b) Resulting training and validation curves for cosine similarity (COS) metric. MSE, MAE, and CS-based loss curves are also displayed.

However, besides the 1296 SD pairs, each SD pair also includes the emissions along four time gates and 12 spectral channels. Hence, a single measurement vector size is 1×62,208. This measurement vector is inputted to the network as shown in Fig. 7(a). The input is then flattened and goes through a combination of dense layers, each one followed by batch normalization and ReLU activations. Dropout is used after multiple dense combinations as a way to reduce the input size to the output size of 1×34,202. Dense layers 2 and 5, as well as dense 1 and 6 are concatenated. Finally, a dense layer of size 1×34,202 is used to map to the output of this same size. The 1×34,202 vector represents the spatial distribution of the contrasts across the total number of nodes. This node vector is in linearized form, however, it can be directly reshaped and plotted into the 3D spatial distribution. The specifics of training are shown in Table 4.

**Table 4:** Details of the training dataset and the MLP architecture used for mesh-based hyperspectral DOT (GPU: NVIDIA GeForce RTX 3090).

Size of dataset	40,000 (1 and 2 embedding)
OPs	$\mu_{a}=0.001$ to $0.07\;{\mathrm{mm}}^{-1}$
	Depth = 2 to 16 mm
	$\mu_{s^{\prime }}=0.1$ to $1.4\;{\mathrm{mm}}^{-1}$
	No. of photons (in simulation) = $10^{7}$
Time to generate dataset	∼8 h
Wavelength channels	740 to 800 nm over 16 slots
Time gates simulated	50r, peak, 80d, 60d
Training/validation split	75/25
Batch size	40
No. of epochs	500
Loss function	MSE and MAE and CS
Optimizer	Adam
Learning rate	$5\times 10^{-5}$
Training time	∼1 h 45 min

For this training, 40,000 samples were generated, containing up to two enhanced EMNIST embeddings, having similar OPs to the ones used in Fig. 3. However, since measurements operate on the time and spectral domains, counts at four time gates were of interest, specifically 50% of the rise (r), the peak intensity time point and 80% and 60% of the decay (d). These percentages are calculated with respect to the peak (maximum) count of the time decay functions. Furthermore, these time gates were obtained per each of the total 16 time decay functions, accounting for each wavelength channel of the detector space. For traditional inverse-based reconstruction, the $W_{\text{matrix}}$ should also represent the four time gates and 16 wavelength channels, as highlighted in Eq. (1). For the loss function, an averaged combination of MSE, mean absolute error (MAE), and cosine similarity (CS) metrics was used. The loss/validation training curves for the network and the curves for CS metric are shown in Fig. 7(b). For testing the trained network, *in silico* samples different from those included in the training data set were used.

**Fig. 8:**
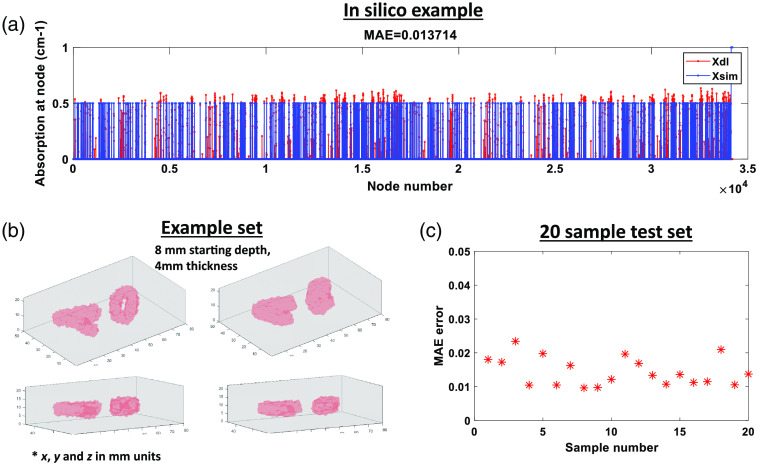
(a) Example *in silico* MLP node output. (b) Reconstruction results with GT shown using the isovolumes. (c) MAE results for a 20 sample test set.

An example of an MLP node vector output is shown in Fig. 8(a) where the Xdl variable represents the output of the network of size 1×34,202, and the Xsim variable represents the ground-truth node vector. For this example a phantom with two embeddings, both with $0.5\;{\mathrm{cm}}^{-1}$ (or $0.05\;{\mathrm{mm}}^{-1}$) of absorption value was used. The MAE between the ground-truth and reconstructed vector equals ∼0.014. Both node vectors, ground-truth, and estimated reconstruction were surface plotted in isovolume form in Fig. 8(b), where the first column represents the GT and the second column the estimated reconstruction. For this phantom, the starting depth is 8 mm with embeddings of 4 mm thickness. The targets’ estimated spatial distribution and absorption value were correctly retrieved as per the calculated MAE. The trained network was tested for 20 random samples with varying depths within 2 to 16 mm. The MAEs for these samples versus GT are shown in Fig. 8(c) where MAEs remain below 0.03. Further testing involves the application of this workflow for experimental settings. However, this material will be presented in a subsequent manuscript. Nevertheless, the usage of a mesh-based approach and a DL framework that can perform end-to-end reconstructions is an important step in applying the proposed optical technique for *in vivo* mouse imaging scenarios.

## 4 Discussion and Conclusions

Herein, voxel (MCX) and mesh (MMC)-based data generation pipelines are proposed to simulate large datasets with nongeometrical randomized variations that can be used to train robust DL end-to-end tomographic/topographic reconstruction architectures. The proposed workflow is based on a single homogeneous photon propagation model and Enhanced EMNIST characters. The data generation pipeline was tested for three different architecture types, addressing a different application within widefield DOT and DOTP. None of these networks have been evaluated for applications other than DOT and DOTP. Further testing and adaptation will be needed if they are to be used for non-DOT applications. However, evaluating the generalization of both the data generation scheme and the networks presented here, beyond applications in DOT and DOTP, is outside the scope of this paper. For this paper, *in silico* measurement vectors were simulated under three different experimental conditions and setups: (1) k-space illumination and gated-ICCD detection setup, (2) widefield illumination and gated ICCD fluorescence lifetime topography system, and (3) a single-pixel widefield detection and illumination DOT system in transmission geometry. DNNs were trained with these data and validated experimentally. Furthermore, the voxel-based workflow built a mesh (MMC)-based data simulation pipeline to reconstruct *in silico* data acquired over up to 12 spectral channels and across timegates. In addition, the rationale behind the proposed data generation pipeline was that even though enhanced EMNIST characters are more complex shapes, using them for DL training of DOT and DOTP architectures would result in more robustly trained networks. In this sense, the networks would be better prepared to tackle complex scenarios of nongeometrically distributed targets while being able to generalize for proper reconstruction of less complex shapes such as spheres, cylinders, etc. Throughout this paper, we have proven that the trained networks could correctly approximate ground-truth absorption values both *in silico* and for experimental phantom validations. Furthermore, despite the simplicity of the reconstructed phantoms, their correct reconstructions had two crucial implications. First, it was proven that, through the proposed data generation pipeline, appropriate W matrices could be accurately simulated to resemble experimental conditions over two different experimental scenarios (K-space DOT, lifetime-based DOTP) as controlled GTs were available. Second, it was shown that even though geometrical shapes such as cylinders were not used during network training, the DNNs could still generalize well to reconstruct them for experimental conditions.

The data generation approach is initially validated through solid phantoms because OPs can be known by design. Furthermore, phantoms can be moved across platforms without disrupting them. For instance, two different experimental setups are used on the same phantom in this work. Such transitions are difficult *in vivo* as absorption properties and targets cannot be easily controlled or known. Though there are many approximations to OPs for mice in literature, it is not guaranteed that differences in external factors, such as the type of mouse strains, will not affect the reported OPs. Hence, starting with simplistic models is fundamental to ensure that the data generation approach is fully working before tackling more complex scenarios where GT settings cannot be validated. However, since *in vivo* imaging is the end goal, future work aims to test the proposed workflows for controlled tumor detection settings *in vivo*. Nevertheless, we need an alternate IACUC approved method to validate *in vivo* OPs within each mouse to proceed appropriately.

Nevertheless, the results indicate that the proposed MCX and MMC data simulation pipelines are well-positioned to train DNNs with high versatility in the presented DOT and DOTP applications. In essence, a robustly trained end-to-end DOT/DOTP architecture can alleviate the burdens resulting from traditionally inverse solving the DOT/DOTP ill-posed inverse problems. Along with the *in vivo* directions discussed above, future work involves using the mesh-based datasets for experimental sets similar to those used throughout this paper for voxel-based pipelines. In addition, the use of a mesh-based workflow, together with time and spectral constraints, should allow for the accurate reconstruction of turbid media of higher complexity, such as in the case of multiexponential parameter retrieval (e.g., Förster resonance energy transfer^30^).

## Code, Data, and Materials Availability

Codes and Example Datasets are available upon author request. They are planned to be made publicly available in due course.
